# Insight into 2α-Chloro-2′(2′,6′)-(Di)Halogenopicropodophyllotoxins Reacting with Carboxylic Acids Mediated by BF_3_·Et_2_O

**DOI:** 10.1038/srep16285

**Published:** 2015-11-17

**Authors:** Lingling Fan, Xiaoyan Zhi, Zhiping Che, Hui Xu

**Affiliations:** 1 Research Institute of Pesticidal Design & Synthesis, College of Sciences, Northwest A&F University, Yangling 712100, Shaanxi Province, P. R. China; 2 State Key Laboratory of Crop Stress Biology for Arid Areas, Northwest A&F University, Yangling 712100, Shaanxi Province, P. R. China

## Abstract

Stereospecific nucleophilic substitution at the C-4α position of 2α-chloro-2′(2′,6′)-(di)halogenopicropodophyllotoxin derivatives with carboxylic acids mediated by BF_3_·Et_2_O was described. Interestingly, this stereoselective products were completely controlled by the reaction time. That is, if the reaction time was prolonged to 24.5–31 h, the resulting compounds were all transformed into the unusual C-ring aromatization products. Additionally, it demonstrated that BF_3_·Et_2_O and reaction temperature were the important factors for C-ring aromatization, and AlCl_3_ could be substituted for BF_3_·Et_2_O as a lewis acid for C-ring aromatization. Halogenation of E-ring of 2β-chloropodophyllotoxins with NCS or NBS also led to the same C-ring aromatization compounds. Especially compounds 5c, 6g and 7b exhibited insecticidal activity equal to that of toosendanin.

Podophyllotoxin (**1**, Fig. 1), an important naturally occurring cyclolignan isolated from the roots and rhizomes of *Podophyllum* species, has been widely used as a lead compound for preparation of potential anticancer agents such as etoposide (VP-16, **2**, Fig. 1), teniposide (VM-26, **3**, Fig. 1), etoposide phosphate, GL331 and TOP53^1^,^2^. On the other hand, compound **1** also exhibited its interesting insecticidal and antifungal activities^3^,^4^,^5^. To find more potent podophyllotoxin derivatives, besides total synthesis of podophyllotoxin and its derivatives^6^,^7^,^8^,^9^,^10^,^11^, structural modifications of them have also attracted much attention in recent years^12^. To solve the instable trans-fused lactone of podophyllotoxin when exposed to mild base, a chlorine/bromine atom was introduced at its C-2α/β position to give 2α/β-halogenopodophyllotoxins (**4**, Fig. 1)^13^,^14^,^15^,^16^. More recently, we found that once a chlorine or bromine atom was firstly introduced at the C-2′ position on the E-ring of podophyllotoxin, 2α-chloro-2′(2′,6′)-(di)halogenopicropodophyllotoxins (**5**, Fig. 1) were then stereoselectively produced, and especially some 4α-acyloxy-2α-chloro-2′(2′,6′)-(di)halogenopicropodophyllotoxins (**6**, Fig. 1) displayed more potent insecticidal activity than toosendanin, a commercial insecticide derived from *Melia azedarach*^17^. Additionally, during the long period of plants evolution, plant secondary metabolites are produced due to the interaction between plants and environment (life and non-life). It is considered that pesticides originating from plant secondary metabolites may result in less or slower resistance development and lower pollution^18^,^19^. Recently, discovery of new insecticidal components directly from plant secondary metabolites, or by using them as the lead-compounds for structural modifications has been one of the important procedures for research and development of new pesticides^20^,^21^,^22^,^23^.

In continuation of our program aimed at the discovery and development of natural-product-based insecticidal agents, we envisaged to prepare a series of 4β-acyloxy-2α-chloro-2′(2′,6′)-(di)halogenopicropodophyllotoxins (**6′**) by S_*N*_1 reaction of **5** with carboxylic acids in the presence of BF_3_·Et_2_O. To our surprise, only compounds **6** were obtained, whereas compounds **6′** were not produced at all. Moreover, the above BF_3_·Et_2_O-mediated reaction was completely controlled by the reaction time. If the reaction time was prolonged, compounds **6** were all transformed into the unusual C-ring aromatization compounds **7**.

## Methods

### Materials and Instruments

All chemical reagents and solvents were of reagent grade or purified according to standard methods before use. Analytical thin-layer chromatography (TLC) and preparative thin-layer chromatography (PTLC) were performed with silica gel plates using silica gel 60 GF_254_ (Qingdao Haiyang Chemical Co., Ltd., Qingdao, China). Silica gel column chromatography was performed with silica gel 200–300 mesh (Qingdao Haiyang Chemical Co., Ltd., Qingdao, China). Melting points were determined on a XT-4 digital melting-point apparatus (Beijing Tech Instrument Co., Ltd., Beijing, China) and were uncorrected. Infrared spectra (IR) were recorded on a Bruker TENSOR 27 spectrometer. Proton nuclear magnetic resonance spectra (^1^H NMR) and carbon nuclear magnetic resonance spectra (^13^C NMR) were recorded in CDCl_3_ or DMSO-*d*_*6*_ on a Bruker Avance DMX 400 or 500 MHz instrument using tetramethylsilane (TMS) as the internal standard. High-resolution mass spectra (HR-MS) were carried out with IonSpec 4.7 Tesla FTMS instrument. Compounds **5a–c** were prepared in the same way as in our previous report^14^.

### General procedure for synthesis of 6a–l

To a mixture of **5a–c** (0.15 mmol) and carboxylic acids (0.18 mmol) in dry CH_2_Cl_2_ (5 mL) at −15 °C, a solution of BF_3_.Et_2_O (0.18 mmol) in dry CH_2_Cl_2_ (5 mL) was added dropwise to keep the temperature below −15 °C. After adding, the reaction temperature was raised from −15 °C to −5 or 0 °C, and the reaction process was checked by TLC analysis. When the reaction was complete, the mixture was diluted by CH_2_Cl_2_ (30 mL), washed by water (20 mL), HCl (0.1 mol/L, 20 mL), 5% aq. NaHCO_3_ (20 mL) and brine (20 mL), dried over anhydrous Na_2_SO_4_, concentrated *in vacuo*, and purified by PTLC to give **6a–l** (40–93% yields) and **7a–c** (6–38% yields) as the white solids. The example data of **6a–c** and **7a–c** are shown as follows, whereas data of **6d–l** can be found in the Supporting Information.

*Data for* **6a**: White solid; m.p. 150–151 °C; [α]^20^_D_ = −78 (*c* 3.5 mg/mL, CHCl_3_); IR cm^−1^ (KBr): 3050, 2936, 1786, 1735, 1487, 1399, 1109, 1016, 867; ^1^H NMR (500 MHz, CDCl_3_) *δ*: 6.68 (s, 1H, H-5), 6.64 (s, 1H, H-8), 6.58 (s, 1H, H-6′), 5.93–5.95 (m, 3H, H-4, OCH_2_O), 5.52 (s, 1H, H-1), 4.80–4.81 (m, 2H, H-11), 3.94 (s, 3H, 3′-OCH_3_), 3.88 (s, 3H, 5′-OCH_3_), 3.76 (s, 3H, 4′-OCH_3_), 2.97–2.98 (m, 1H, H-3), 2.16 (s, 3H, COCH_3_); ^13^C NMR (125 MHz, CDCl_3_) *δ*: 171.9, 170.6, 152.0, 149.9, 149.2, 148.2, 142.6, 132.1, 130.8, 123.9, 122.0, 108.8, 108.6, 108.3, 101.7, 75.1, 73.2, 66.8, 61.1, 61.1, 56.1, 49.3, 44.7, 21.1; HRMS (ESI): Calcd for C_24_H_22_O_9_Cl_2_Na ([M + Na]^+^), 547.0533; found, 547.0529.

*Data for* **6b**: White solid; m.p. 179–180 °C; [α]^20^_D_ = −80 (*c* 2.9 mg/mL, CHCl_3_); IR cm^−1^ (KBr): 3057, 2935, 1788, 1724, 1486, 1400, 1108, 1033, 868; ^1^H NMR (400 MHz, CDCl_3_) *δ*: 6.67 (s, 1H, H-5), 6.62 (s, 1H, H-8), 6.57 (s, 1H, H-6′), 5.93–5.94 (m, 3H, OCH_2_O, H-4), 5.51 (s, 1H, H-1), 4.80–4.81 (m, 2H, H-11), 3.93 (s, 3H, 3′-OCH_3_), 3.88 (s, 3H, 5′-OCH_3_), 3.75 (s, 3H, 4′-OCH_3_), 2.96–2.97 (m, 1H, H-3), 2.35–2.42 (m, 2H, CH_3_CH_2_), 1.20 (t, *J* = 7.6 Hz, 3H, CH_3_CH_2_); ^13^C NMR (100 MHz, CDCl_3_) *δ*: 174.2, 171.9, 152.1, 150.0, 149.2, 148.2, 142.8, 132.2, 130.7, 124.2, 122.1, 108.8, 108.6, 108.5, 101.7, 74.9, 73.1, 66.8, 61.1, 61.1, 56.2, 49.4, 44.7, 27.5, 9.0; HRMS (ESI): Calcd for C_25_H_24_O_9_Cl_2_Na ([M + Na]^+^), 561.0689; found, 561.0691.

*Data for* **6c**: White solid; m.p. 79–80 °C; [α]^19^_D_ = −66 (*c* 3.6 mg/mL, CHCl_3_); IR cm^−1^ (KBr): 3062, 2936, 1789, 1737, 1486, 1399, 1110, 1034, 864, 697; ^1^H NMR (400 MHz, CDCl_3_) *δ*: 7.27–7.39 (m, 5H), 6.67 (s, 1H, H-5), 6.57 (s, 1H, H-8), 6.51 (s, 1H, H-6′), 5.93 (d, *J* = 1.2 Hz, 2H, OCH_2_O), 5.89 (d, *J* = 3.2 Hz, 1H, H-4), 5.50 (s, 1H, H-1), 4.77–4.78 (m, 2H, H-11), 3.93 (s, 3H, 3′-OCH_3_), 3.89 (s, 3H, 5′-OCH_3_), 3.75 (s, 3H, 4′-OCH_3_), 3.67 (d, *J* = 6.8 Hz, 2H, PhCH_2_), 2.99–3.00 (m, 1H, H-3); ^13^C NMR (100 MHz, CDCl_3_) *δ*: 171.8, 171.6, 152.0, 150.0, 149.2, 148.2, 142.8, 132.6, 132.1, 130.6, 129.0, 128.9, 127.7, 123.9, 122.1, 108.7, 108.6, 108.4, 101.7, 75.5, 72.9, 66.7, 61.1, 61.1, 56.3, 49.2, 44.6, 41.1; HRMS (ESI): Calcd for C_30_H_26_O_9_Cl_2_Na ([M + Na]^+^), 623.0846; found, 623.0849.

*Data for* **7a**: White solid; m.p. 168–169 °C; IR cm^−1^ (KBr): 3064, 2934, 2845, 1762, 1466, 1109, 1036, 1012, 883; ^1^H NMR (500 MHz, DMSO-*d*_*6*_) *δ*: 8.01 (s, 1H), 7.56 (s, 1H), 6.77 (s, 1H), 6.71 (s, 1H), 6.20 (s, 2H, OCH_2_O), 5.44–5.53 (m, 2H, H-11), 3.90 (s, 3H, OCH_3_), 3.87 (s, 3H, OCH_3_), 3.74 (s, 3H, OCH_3_); ^13^C NMR (125 MHz, DMSO-*d*_*6*_) *δ*: 168.8, 151.8, 149.7, 149.1, 148.6, 142.3, 140.0, 135.2, 134.2, 129.4, 128.7, 120.1, 118.8, 118.3, 109.9, 130.7, 102.1, 101.5, 68.2, 60.9, 60.6, 56.0; HRMS (ESI): Calcd for C_22_H_17_O_7_ClNa ([M + Na]^+^), 451.0555; found, 451.0570.

*Data for* **7b**: White solid; m.p. 218–219 °C; IR cm^−1^ (KBr): 3052, 2942, 2911, 1745, 1467, 1207, 1010, 895; ^1^H NMR (500 MHz, DMSO-*d*_*6*_) *δ*: 8.05 (s, 1H), 7.58 (s, 1H), 6.75 (s, 1H), 6.22 (s, 2H, OCH_2_O), 5.53 (s, 2H, H-11), 4.02 (s, 3H, 4′-OCH_3_), 3.89 (s, 6H, 3′, 5′-OCH_3_); ^13^C NMR (125 MHz, DMSO-*d*_*6*_) *δ*: 168.8, 149.9, 149.1, 148.8, 147.5, 140.1, 134.5, 132.3, 128.5, 128.2, 122.7, 120.8, 118.8, 103.9, 102.3, 100.7, 68.5, 61.2, 61.0; HRMS (ESI): Calcd for C_22_H_16_O_7_Cl_2_Na ([M + Na]^+^), 485.0165; found, 485.0170.

*Data for* **7c**: White solid; m.p. 237–238 °C; IR cm^−1^ (KBr): 3062, 2920, 1758, 1619, 1460, 1249, 1101, 895. ^1^H NMR (400 MHz, CDCl_3_) *δ*: 7.74 (s, 1H), 7.22 (s, 1H), 6.84 (s, 1H), 6.59 (s, 1H), 6.09 (s, 2H, OCH_2_O), 5.35–5.45 (m, 2H, H-11), 4.01 (s, 3H, OCH_3_), 3.98 (s, 3H, OCH_3_), 3.79 (s, 3H, OCH_3_); ^13^C NMR (100 MHz, CDCl_3_) *δ*: 169.2, 152.7, 151.1, 150.1, 148.9, 143.0, 139.7, 138.4, 134.6, 131.5, 129.7, 119.6, 119.3, 110.3, 109.7, 103.8, 103.0, 101.9, 68.3, 61.3, 61.2, 56.1; HRMS (ESI): Calcd for C_22_H_18_O_7_Br ([M + H]^+^), 473.0230; found, 473.0235.

### Biological assay^17^

The insecticidal activity of **5a–c**, **6a–l** and **7a–c** against the pre-third-instar larvae of *Mythimna separata* Walker was assessed by leaf-dipping method. For each compound, 30 larvae (10 larvae per group) were used. Acetone solutions of compounds **5a–c**, **6a–l**, **7a–c**, and toosendanin (used as a positive control) were prepared at the concentration of 1 mg/mL. Fresh wheat leaves were dipped into the corresponding solution for 3 s, then taken out, and dried in a room. Leaves treated with acetone alone were used as a blank control group. Several treated leaves were kept in each dish, where every 10 larvae were raised. If the treated leaves were consumed, additional treated leaves were added to the dish. After 48 h, untreated fresh leaves were added to all dishes until adult emergence. The experiment was carried out at 25 ± 2 °C and relative humidity (RH) 65–80%, and on 12 h/12 h (light/dark) photoperiod. The insecticidal activity of the tested compounds against the pre-third-instar larvae of *M. separata* was calculated by the following formula:


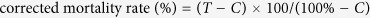


where *T* is the mortality rate in the treated group expressed as a percentage and *C* is the mortality rate in the untreated group expressed as a percentage.

## Results and Discussion

**Figure 2:**
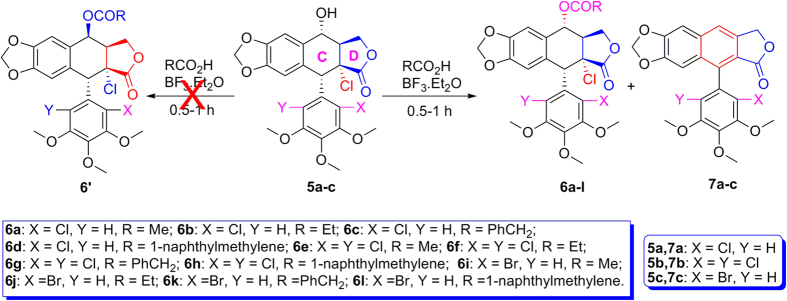
Compounds 5a–c reacting with carboxylic acids in the presence of BF_3_·Et_2_O.

**Figure 3:**
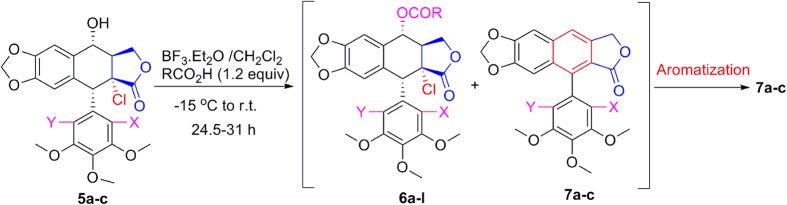
Compounds 5a-c reacting with carboxylic acids in the presence of BF_3_·Et_2_O for a prolonged time.

**Figure 4:**
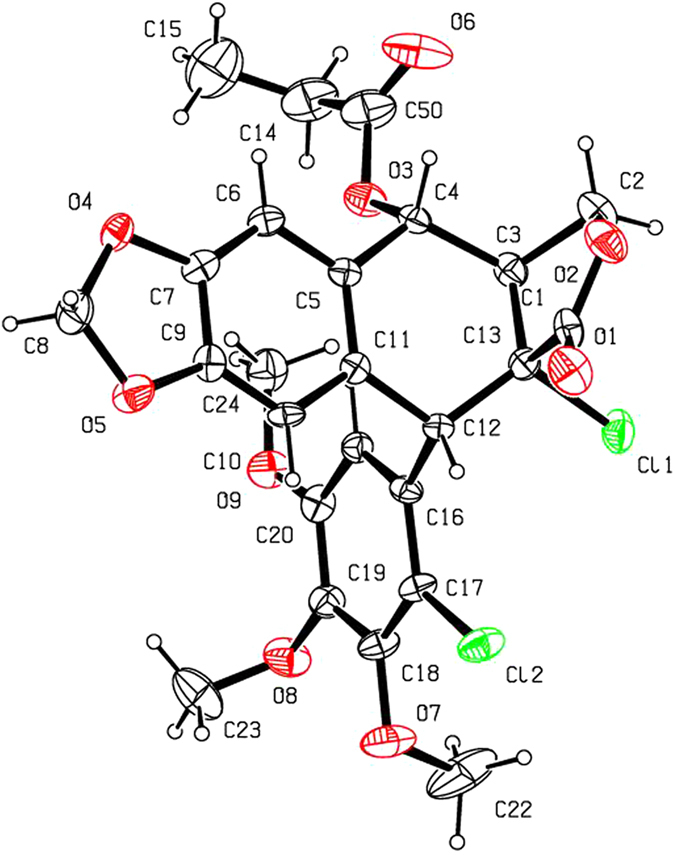
X-ray crystal structure of compound 6b. Drawing by Hui Xu.

**Figure 5:**
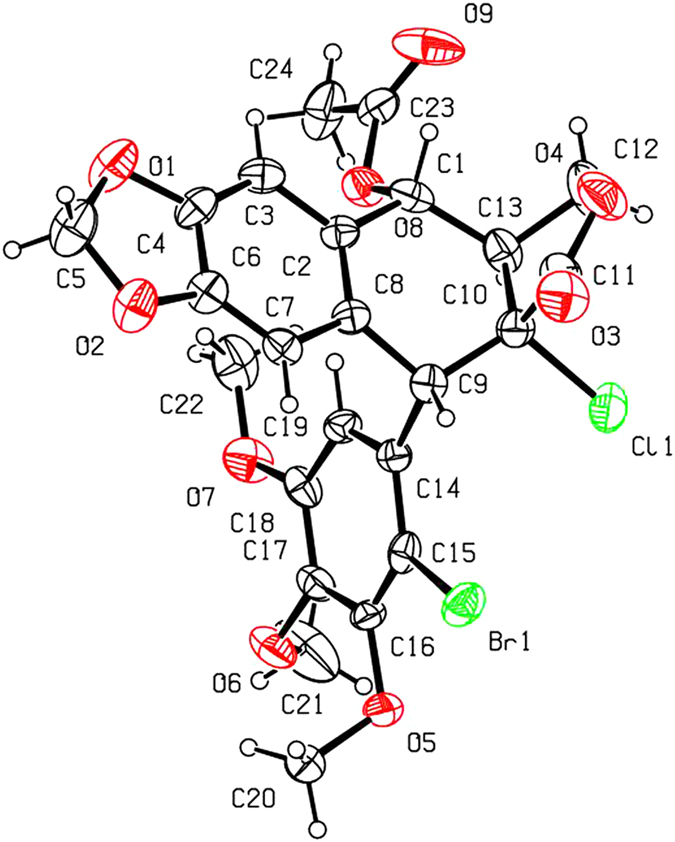
X-ray crystal structure of compound 6i. Drawing by Hui Xu.

**Figure 6:**
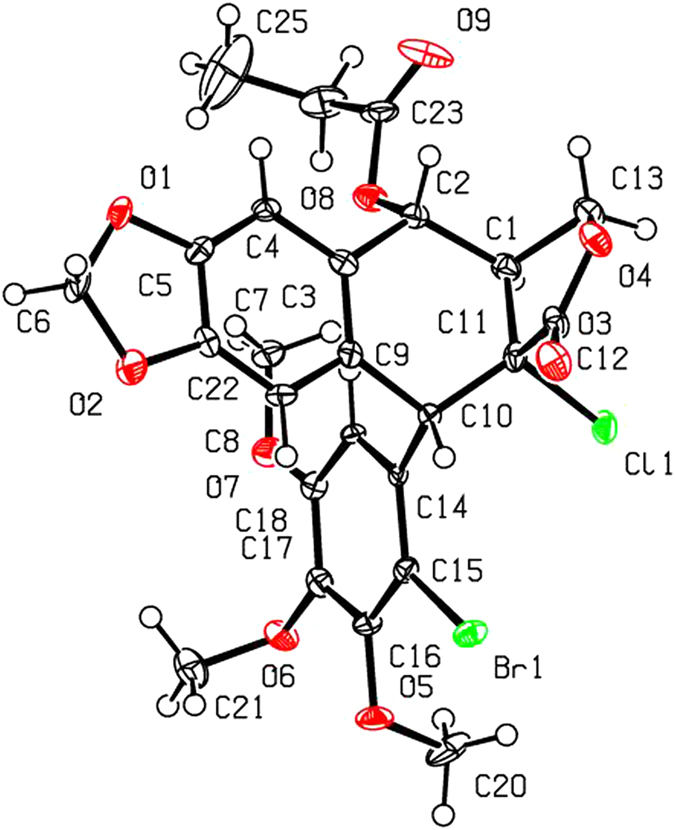
X-ray crystal structure of compound 6j. Drawing by Hui Xu.

**Figure 7:**
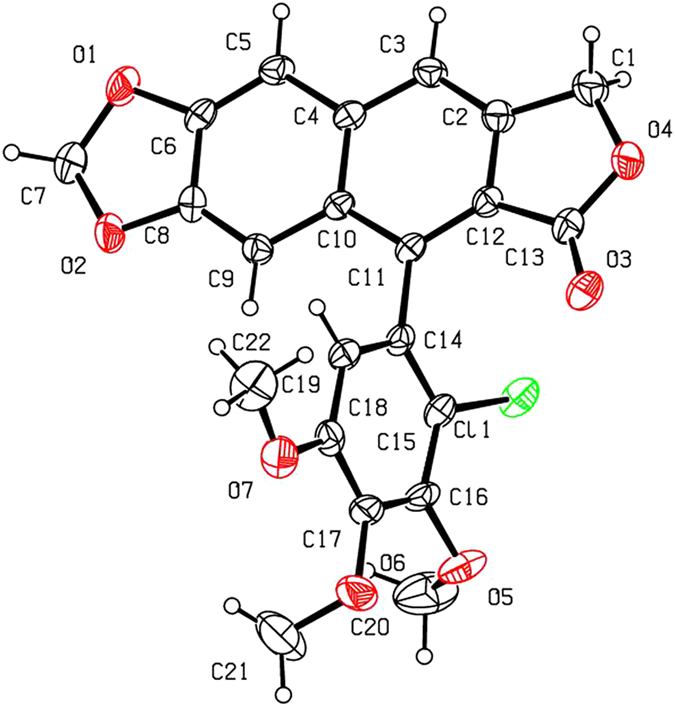
X-ray crystal structure of compound 7a. Drawing by Hui Xu.

**Figure 8:**
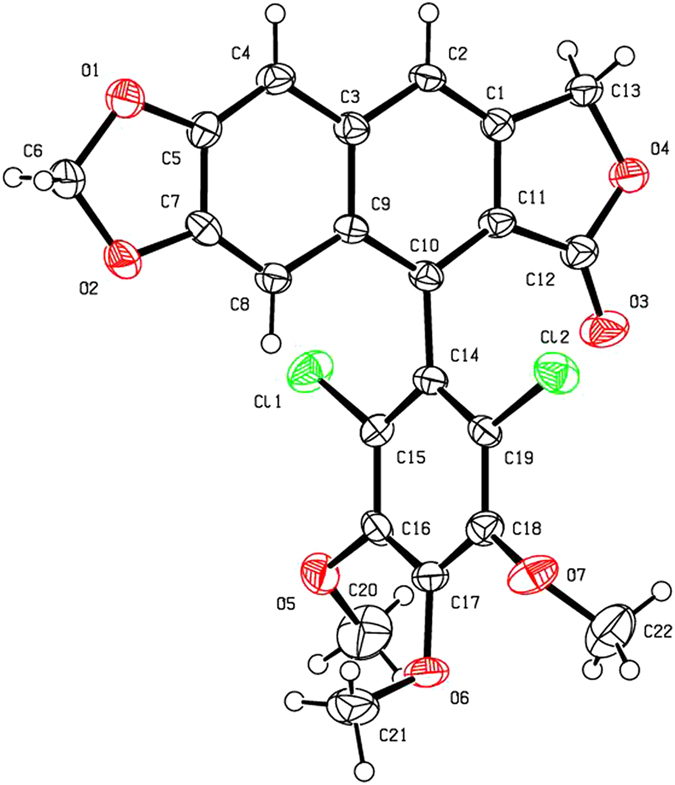
X-ray crystal structure of compound 7b. Drawing by Hui Xu.

**Figure 9:**
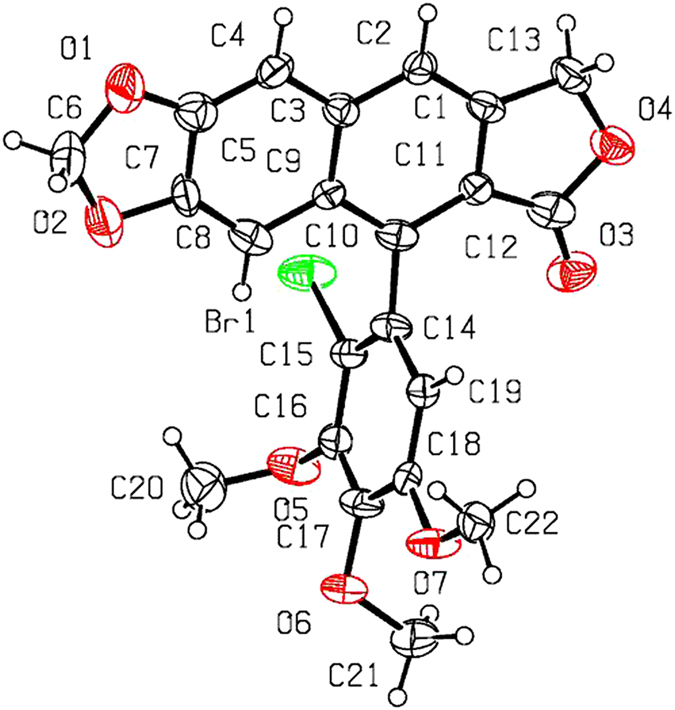
X-ray crystal structure of compound 7c. Drawing by Hui Xu.

As shown in Fig. 2 and Table 1, when compounds **5a–c** were allowed to react with carboxylic acids in the presence of BF_3_·Et_2_O at −15 °C to −5 °C for 0.5 h (or at −15 °C to 0 °C for 1 h), the expected compounds **6′** were not obtained, whereas compounds **6a–l** were stereoselectively afforded in 40–93% yields. Meanwhile, the corresponding by-products **7a–c**, containing the aromatized C-ring, were also afforded. Interestingly, the amount of **7a–c** would be increased with the advance of time; on the contrary, the amount of **6a–l** would be decreased accordingly. Subsequently, compounds **5a–c** reacting with carboxylic acids in the presence of BF_3_·Et_2_O for a prolonged time was investigated as described in Fig. 3 and Table 2. It generally took 24.5–31 h for **6a–l** all transformed into the corresponding compounds **7a–c** in 40–99% yields. The assignment of configuration of acyloxy at the C-4 position of **6c**, **6d**, **6k** and **6l** (containing the *cis*-lactone, and an halogen atom on the E-ring) was according to our previous research results: if *J*_3.4_ ≈ 2.0 Hz, it indicates that H-3 and H-4 is *trans* relationship, that is, the substituent at the C-4 position of picropodophyllotoxin is α configuration^15^. The *J*_3.4_ values of H-4 of **6c**, **6d**, **6k** and **6l** were 3.2 Hz, therefore, the substituents at the C-4 position of **6c**, **6d**, **6k** and **6l** were α configuration. Because the NMR spectra of **6e–h** (bearing the *cis*-lactone, and two chlorine atoms on the E-ring) were the same as those of **6e′-h′** (compounds **6e′–h′** were prepared from **5b** in the presence of DCC and DMAP with acetic acid, propionic acid, phenylacetic acid, and 1-naphthylacetic acid, respectively. See Supporting Information), so the configuration of acyloxy at the C-4 position of **6e–h** was α. The configuration of **6b**, **6i**, **6j**, and **7a–c** was confirmed by the X-ray crystallography (Figs 4, 5, 6, 7, 8, 9). Six crystallographic data (excluding structure factors) for the structures of **6b**, **6i**, **6j**, and **7a–c** have been deposited with the Cambridge Crystallographic Data Centre as supplementary publication number CCDC 918891, 922185, 922186, 918697, 918890, and 922184, respectively. Copies of the data can be obtained, free of charge, on application to CCDC, 12 Union Road, Cambridge CB21EZ, UK [fax: + 44 (0)1223 336033 or e-mail: deposit@ccdc.cam.ac.uk]. It clearly demonstrated that the propionyloxy of **6b**, acetyloxy of **6i**, and propionyloxy of **6j** at the C-4 position all were present in α configuration. The C-ring of **7a–c** was all aromatized. Meanwhile, based on the X-ray crystallography (Figs 4, 5, 6), if the acyloxy group at the C-4 position adopted β configuration, big steric effects would be observed between the lactone and the acyloxy group. Hence the acyloxy group at the C-4 position of **6a–l** were all present in α configuration.

**Figure 10:**
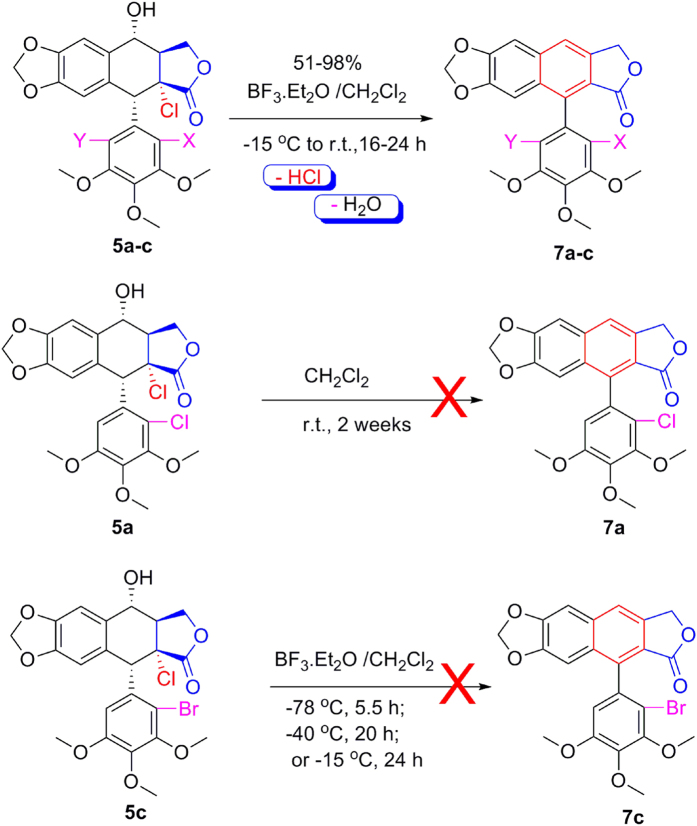
Investigation of 5a–c mediated by BF_3_·Et_2_O or not.

Furthermore, compounds **5a**, **5b** or **5c** with BF_3_·Et_2_O in CH_2_Cl_2_ was also examined. As described in Fig. 10, when the reaction time was prolonged to 16–24 h, compounds **5a–c** were smoothly transformed into the corresponding compounds **7a-c** in 51–98% yields by C-ring aromatization. However, when only compound **5a** in CH_2_Cl_2_ was stirred at room temperature for two weeks, except **5a**, no product was produced. Additionally, when compound **5c** with BF_3_·Et_2_O in CH_2_Cl_2_ was stirred at −78 °C for 5.5 h, −40 °C for 20 h, or −15 °C for 24 h, except **5c**, the target compound **7c** was not obtained. It demonstrated that BF_3_·Et_2_O and reaction temperature were the important factors for C-ring aromatization of **5a–c**.

**Figure 11:**
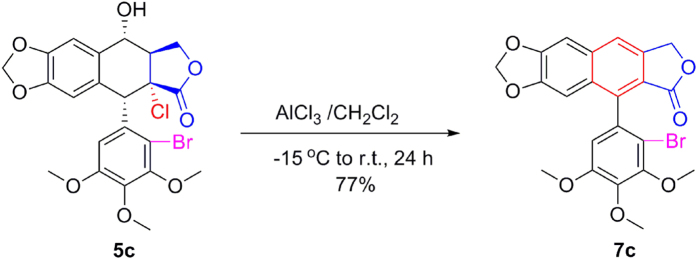
Investigation of 5c mediated by AlCl_3_.

**Figure 12:**
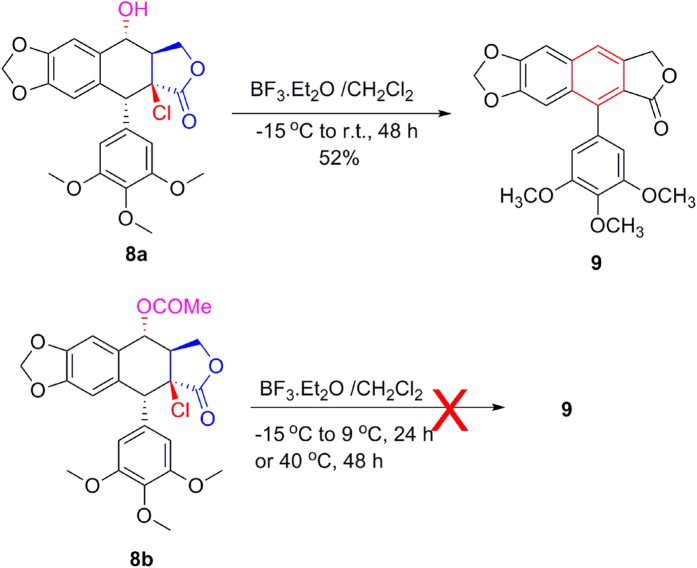
Investigation of 8a and 8b mediated by BF_3_·Et_2_O.

Subsequently, as shown in Fig. 11, when compound **5c** with AlCl_3_ in CH_2_Cl_2_ was stirred at −15 °C to r.t. for 24 h, compound **7c** was obtained in 77% yield. It suggested that AlCl_3_ could be substituted for BF_3_·Et_2_O as a lewis acid for C-ring aromatization. It might involve a BF_3_·Et_2_O-promoted dehydrochlorination, followed by a BF_3_·Et_2_O-promoted dehydration or dehydroacylation. Meanwhile, as shown in Fig. 12, when 2β-chloropodophyllotoxin (**8a**) in the presence of BF_3_·Et_2_O was stirred at −15 °C to r.t. for 48 h, C-ring aromatization product **9** was obtained in 52% yield (see Supporting Information). However, 4α-acetyloxy-2β-chloropodophyllotoxin (**8b**) in the presence of BF_3_·Et_2_O was stirred at −15 °C to 9 °C for 24 h, or at 40 °C for 48 h, the proposed product **9** was not detected.

**Figure 13:**
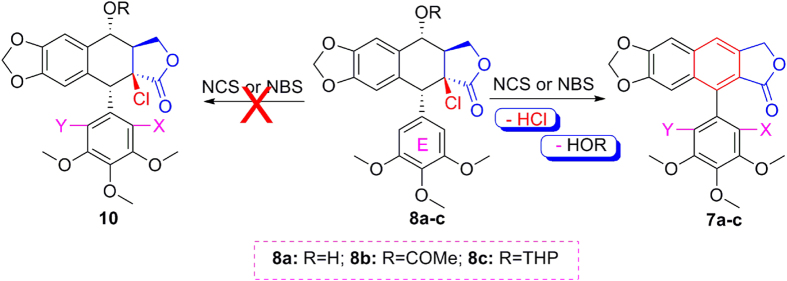
2β-Chloropodophyllotoxins (8a–c) reacting with NCS or NBS.

On the other hand, as shown in Fig. 13, we envisaged whether 2β-chloro-2′(2′,6′)-(di)halogenopodophyllotoxin derivatives (**10**, the diastereoisomers of **6**), could be prepared by direct halogenation of E-ring of 2β-chloropodophyllotoxins. As described in Table 3, the reaction of 2β-chloropodophyllotoxins (**8a–c**) with NCS or NBS was further examined. When compound **8a** reacted with 1.1 equiv of NCS at 25 °C for 24 h, no product was obtained (entry 1). When the reaction temperature was raised to 30 °C, the target E-ring halogenation product was not produced; interestingly, whereas the C-ring aromatization compounds such as **7a** and **7b** were obtained in 35% and 15% yields, respectively (entry 2). And when at 40 °C for 4 h, compounds **7a** and **7b** were obtained in 54% and 12% yields, respectively (entry 3). However, when the reaction temperature was raised to 50 °C, the yields of **7a** and **7b** were not increased (entry 4). When compound **8a** reacted with 2.2 equiv of NCS at 40 °C for 11 h, only **7b** was obtained in 52% yield (entry 5). Similarly, when compound **8a** reacted with 1.1 equiv of NBS at 40 °C for 5 h, compound **7c** was obtained in 50% yield (entry 7); when compound **8c** reacted with 1.1 equiv of NCS at 40 °C for 19 h, compounds **7a** and **7b** were obtained in 34% and 25% yields, respectively (entry 12). But when compound **8b** reacted with NCS or NBS, the reaction temperature should be raised. For example, when compound **8b** reacted with 1.1 equiv of NCS at 40 °C for 48 h, compound **7a** was obtained only in 12% yield (entry 8); whereas the reaction temperature was raised to 60 °C for 24 h, compound **7a** was obtained in 94% (entry 9). When compound **8b** reacted with 2.2 equiv of NCS for 17 h or 1.1 equiv of NBS for 24 h at 60 °C, compounds **7b** and **7c** were obtained in 87% and 91% yields, respectively (entries 10 and 11). All in all, the reaction temperature was very important for 2β-chloropodophyllotoxins reacting with NCS or NBS to give **7a–c**. According to our previous results^14^, reaction of **8a–c** with NCS or NBS might involve E-ring halogenation of 2β-chloropodophyllotoxins, followed by the dehydrochlorination and dehydration or dehydroacylation.

Finally, compounds **5a–c**, **6a–l**, and **7a–c** were evaluated as insecticidal agents against the pre-third-instar larvae of oriental armyworm, *Mythimna separata* (Walker) at the concentration of 1 mg/mL. Among **5a–c** and **6a–l**, as described in our previous paper^17^, only compounds **5c** and **6g** exhibited insecticidal activity equal to that of toosendanin, and the final corrected mortality rates of **5c**, **6g** and toosendanin were 51.9%, 55.6% and 51.9%, respectively. The final corrected mortality rates of **1** and **7a–c** were 37%, 40.7%, 51.9%, and 37%, respectively.

## Conclusion

In summary, we have developed a BF_3_·Et_2_O-mediated stereoselective synthesis 4α-acyloxy-2α-chloro-2′(2′,6′)-(di)halogenopicropodophyllotoxin derivatives, which was completely controlled by the reaction time. If the reaction time was prolonged to 24.5–31 h, the target compounds were all transformed into the C-ring aromatization compounds. Additionally, it demonstrated that BF_3_·Et_2_O and reaction temperature were the important factors for C-ring aromatization, and AlCl_3_ could be substituted for BF_3_·Et_2_O as a lewis acid for C-ring aromatization. However, in the presence of NCS or NBS, 2β-chloropodophyllotoxins could also be transformed into the same C-ring aromatization compounds. Notably, compounds **5c**, **6g** and **7b** exhibited insecticidal activity equal to that of toosendanin.

## Additional Information

**How to cite this article**: Fan, L. *et al.* Insight into 2α-Chloro-2′(2′,6′)-(Di)Halogenopicropodophyllotoxins Reacting with Carboxylic Acids Mediated by BF_3_·Et_2_O. *Sci. Rep.* **5**, 16285; doi: 10.1038/srep16285 (2015).

## Supplementary Material

Supplementary Information

## Figures and Tables

**Figure 1:**
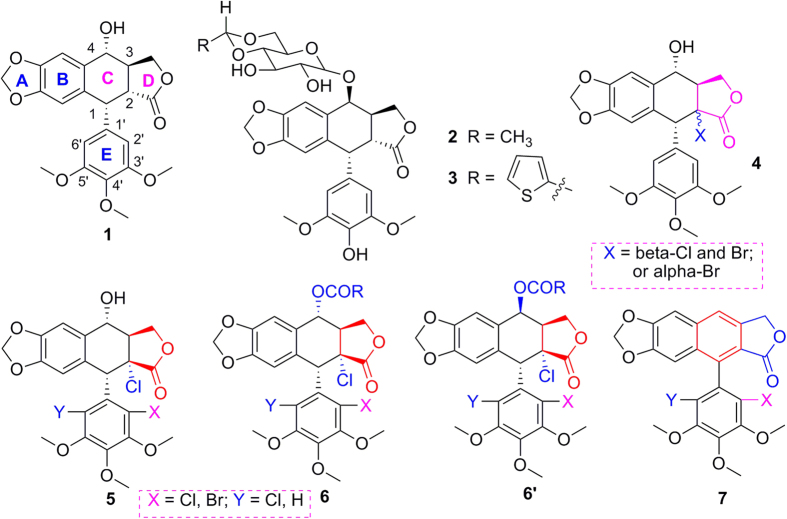
Chemical structures of podophyllotoxin (1) and its derivatives (2–7).

**Table 1:** Investigation of 5a-c Reacting with Carboxylic Acids Mediated by BF_3_·Et_2_O.

Entry	Compound	Acid	*T*[°C]	*t*[h]	Isolated yield [%]	
					6	7
1	**5a**	acetic acid	−15 ~ −5	0.5	**6a** (75)	**7a** (27)
2	**5a**	propionic acid	−15 ~ −5	0.5	**6b** (47)	**7a** (27)
3	**5a**	phenylacetic acid	−15 ~ −5	0.5	**6c** (66)	**7a** (21)
4	**5a**	1-naphthylacetic acid	−15 ~ −5	0.5	**6d** (53)	**7a** (22)
5	**5b**	acetic acid	−15 ~ −5	0.5	**6e** (64)	**7b** (14)
6	**5b**	propionic acid	−15 ~ −5	0.5	**6f** (40)	**7b** (23)
7	**5b**	phenylacetic acid	−15 ~ −5	0.5	**6g** (78)	**7b** (11)
8	**5b**	1-naphthylacetic acid	−15 ~ 0	1	**6h** (50)	**7b** (38)
9	**5c**	acetic acid	−15 ~ −5	0.5	**6i** (93)	**7c** (trace)
10	**5c**	propionic acid	−15 ~ −5	0.5	**6j** (66)	**7c** (6)
11	**5c**	phenylacetic acid	−15 ~ 0	1	**6k** (72)	**7c** (27)
12	**5c**	1-naphthylacetic acid	−15 ~ 0	1	**6l** (50)	**7c** (29)

**Table 2:** Compounds 5a–c Reacting with Carboxylic Acids Mediated by BF_3_·Et_2_O for a Prolonged Time.

Entry	Compound	Acid	*t*[h]	Isolated yield [%]
1	**5a**	acetic acid	25.5	**7a** (75)
2	**5a**	propionic acid	25.5	**7a** (80)
3	**5a**	phenylacetic acid	30.5	**7a** (47)
4	**5a**	1-naphthylacetic acid	24.5	**7a** (93)
5	**5b**	acetic acid	30.5	**7b** (90)
6	**5b**	propionic acid	28.5	**7b** (40)
7	**5b**	phenylacetic acid	24.5	**7b** (41)
8	**5b**	1-naphthylacetic acid	26	**7b** (91)
9	**5c**	acetic acid	25.5	**7c** (99)
10	**5c**	propionic acid	30.5	**7c** (50)
11	**5c**	phenylacetic acid	25	**7c** (78)
12	**5c**	1-naphthylacetic acid	31	**7c** (64)

**Table 3:** Investigation of 2β-Chloropodophyllotoxins (8a–c) Reacting with NCS or NBS.

Entry	Compound	Amount of NCS orNBS	*T*[°C]	*t*[h]	Isolated yield [%]
1	**8a**	NCS (1.1 equiv)	25	24	**7a** (0)
2	**8a**	NCS (1.1 equiv)	30	7	**7a** (35) + **7b** (15)
3	**8a**	NCS (1.1 equiv)	40	4	**7a** (54) + **7b** (12)
4	**8a**	NCS (1.1 equiv)	50	4	**7a** (40) + **7b** (7)
5	**8a**	NCS (2.2 equiv)	40	11	**7b** (52)
6	**8a**	NBS (1.1 equiv)	25	24	**7c** (0)
7	**8a**	NBS (1.1 equiv)	40	5	**7c** (50)
8	**8b**	NCS (1.1 equiv)	40	48	**7a** (12)
9	**8b**	NCS (1.1 equiv)	60	24	**7a** (94) + **7b** (trace)
10	**8b**	NCS (2.2 equiv)	60	17	**7b** (87)
11	**8b**	NBS (1.1 equiv)	60	24	**7c** (91)
12	**8c**	NCS (1.1 equiv)	40	19	**7a** (34) + **7b** (25)
