# Supplementary material for: Insight into 2α-Chloro-2′(2′,6′)-(Di)Halogenopicropodophyllotoxins Reacting with Carboxylic Acids Mediated by BF3·Et2O
Source: Sci Rep. 2015 Nov 17;5:16285. doi: 10.1038/srep16285 (PMC4648097; doi:10.1038/srep16285)

**Supporting Information**

**Insight into 2α-Chloro-2′(2′,6′)-(Di)Halogenopicropodophyllotoxins Reacting with Carboxylic Acids Mediated by BF_3_^.^Et_2_O**

Lingling Fan^1,*^, Xiaoyan Zhi^1,*^, Zhiping Che^1^ & Hui Xu^1,2^

^1^Research Institute of Pesticidal Design & Synthesis, College of Sciences, Northwest A&F University, Yangling 712100, Shaanxi Province, P. R. China.

^2^State Key Laboratory of Crop Stress Biology for Arid Areas, Northwest A&F University, Yangling 712100, Shaanxi Province, P. R. China.

Correspondence and requests for materials should be addressed to H.X. ([orgxuhui@nwsuaf.edu.cn](mailto:orgxuhui@nwsuaf.edu.cn)); Telephone: +86(0)29-87091952; Fax: +86(0)29-87091952.

^*^These authors contributed equally to this work.

**CONTENTS**

1. Synthesis and characterization ……………………………………………….**2-7**

2. Comparison of **6e-h** and **6e′-h′** about NMR spectra ……..…….………………………..**8-11**

3. Copies of ^1^H NMR and ^13^C NMR spectra ……..…….………………………**12-42**

**1. Synthesis and characterization**

**1.1. Characterization of 6d-l**

*Data for* **6d**: Yield: 85%, white solid; m.p. 92-94 ^o^C; [α]^20^_D_ = －56 (*c* 3.2 mg/mL, CHCl_3_); IR cm^-1^ (KBr): 3047, 2935, 1789, 1736, 1485, 1398, 1110, 1035, 863, 780; ^1^H NMR (400 MHz, CDCl_3_) *δ*: 7.90-7.93 (m, 2H), 7.84-7.86 (m, 1H), 7.53-7.56 (m, 2H), 7.38-7.47 (m, 2H), 6.68 (s, 1H, H-5), 6.55 (s, 1H, H-8), 6.43 (s, 1H, H-6′), 5.92 (dd, *J* = 6.8, 1.2 Hz, 2H, OCH_2_O), 5.87 (d, *J* = 3.2 Hz, 1H, H-4), 5.50 (s, 1H, H-1), 4.73 (d, *J* = 2.8 Hz, 2H, H-11), 4.12 (d, *J* = 7.2 Hz, 2H, C_10_H_7_CH_2_), 3.94 (s, 3H, 3′-OCH_3_), 3.89 (s, 3H, 5′-OCH_3_), 3.72 (s, 3H, 4′-OCH_3_), 2.99 (dd, *J* = 6.0, 2.8 Hz, 1H, H-3); ^13^C NMR (100 MHz, CDCl_3_) *δ*: 171.8, 171.7, 152.0, 150.0, 149.2, 148.2, 142.8, 133.9, 132.2, 131.7, 130.6, 129.3, 129.1, 128.7, 127.9, 126.8, 126.1, 125.5, 123.9, 123.0, 122.1, 108.7, 108.3, 101.7, 75.5, 72.8, 66.7, 61.14, 61.12, 56.3, 49.2, 44.6, 39.0; HRMS (ESI): Calcd for C_34_H_28_O_9_Cl_2_Na ([M+Na]^+^), 673.1002; found, 673.0973.

*Data for* **6e**: Yield: 90%, white solid; m.p. 92-93 ^o^C; [α]^20^_D_ = －86 (*c* 3.3 mg/mL, CHCl_3_); IR cm^-1^ (KBr): 3076, 2938, 1791, 1742,1486, 1408, 1235, 1021, 873; ^1^H NMR (400 MHz, CDCl_3_) *δ*: 6.51 (s, 1H, H-5), 6.23 (s, 1H, H-8), 6.07 (s, 1H, H-1), 5.92-5.93 (m, 3H, OCH_2_O, H-4), 4.76-4.78 (m, 1H, H-11), 4.57-4.59 (m, 1H, H-11), 3.98 (s, 3H, 3′-OCH_3_), 3.96 (s, 3H, 5′-OCH_3_), 3.83 (s, 3H, 4′-OCH_3_), 3.39-3.41 (m, 1H, H-3), 2.24 (s, 3H, COCH_3_); ^13^C NMR (100 MHz, CDCl_3_) *δ*: 172.8, 171.1, 149.3, 148.9, 148.1, 147.6, 147.4, 133.3, 127.5, 125.9, 125.5, 107.8, 105.7, 101.5, 71.2, 70.9, 64.9, 61.3, 61.28, 61.20, 48.9, 44.1, 21.1; HRMS (ESI): Calcd for C_24_H_21_O_9_Cl_3_Na ([M+Na]^+^), 581.0143; found, 581.0150.

*Data for* **6f**: Yield: 61%, white solid; m.p. 84-86 ^o^C; [α]^20^_D_ = －107 (*c* 2.8 mg/mL, CHCl_3_); IR cm^-1^ (KBr): 3076, 2981, 2939, 1790, 1740, 1485, 1408, 1237, 1168, 1031, 869; ^1^H NMR (400 MHz, CDCl_3_) *δ*: 6.49 (s, 1H, H-5), 6.23 (s, 1H, H-8), 6.07 (s, 1H, H-1), 5.90-5.93 (m, 3H, OCH_2_O, H-4), 4.75 (dd, *J* = 7.2, 3.2 Hz, 1H, H-11), 4.58 (d, *J* = 7.6 Hz, 1H, H-11), 3.98 (s, 3H, 3′-OCH_3_), 3.96 (s, 3H, 5′-OCH_3_), 3.83 (s, 3H, 4′-OCH_3_), 3.382 (dd, *J* = 5.6, 2.8 Hz, 1H, H-3), 2.49-2.52 (m, 2H, COCH_2_CH_3_), 1.23 (t, *J* = 6.0 Hz, 3H, COCH_2_CH_3_); ^13^C NMR (100 MHz, CDCl_3_) *δ*: 174.4, 172.8, 149.3, 149.0, 148.1, 147.6, 147.4, 133.3, 127.5, 126.1, 125.5, 107.8, 105.8, 101.4, 71.3, 70.9, 65.0, 61.3, 61.2, 61.1, 49.1, 44.1, 27.7, 9.1; HRMS (ESI): Calcd for C_25_H_23_O_9_Cl_3_Na ([M+Na]^+^), 595.0299; found, 595.0300.

*Data for* **6g**: Yield: 71%, white solid; m.p. 78-80 ^o^C; [α]^20^_D_ = －95 (*c* 2.8 mg/mL, CHCl_3_); IR cm^-1^ (KBr): 3063, 2937, 1790, 1738, 1485, 1408, 1238, 1167, 1024, 699; ^1^H NMR (400 MHz, CDCl_3_) *δ*: 7.31-7.378 (m, 5H), 6.34 (s, 1H, H-5), 6.21 (s, 1H, H-8), 6.05 (s, 1H, H-1), 5.885 (dd, *J* = 10.0, 1.2 Hz, 2H, OCH_2_O), 5.86 (d, *J* = 7.2 Hz, 1H, H-4), 4.65 (dd, *J* = 9.2, 4.0 Hz, 1H, H-11), 4.46 (d, *J* = 9.6 Hz, 1H, H-11), 3.97 (s, 3H, 3′-OCH_3_), 3.95 (s, 3H, 5′-OCH_3_), 3.83 (s, 3H, 4′-OCH_3_), 3.76 (s, 2H, PhCH_2_), 3.31 (dd, *J* = 7.2, 3.6 Hz, 1H, H-3); ^13^C NMR (100 MHz, CDCl_3_) *δ*: 172.7, 171.6, 149.2, 148.9, 148.1, 147.6, 147.3, 133.2, 133.1, 129.2, 128.8, 127.6, 127.5, 127.5, 125.8, 125.5, 107.7, 105.8, 101.4, 71.6, 71.1, 64.9, 61.3, 61.2, 61.1, 49.0, 44.1, 41.5; HRMS (ESI): Calcd for C_30_H_25_O_9_Cl_3_Na ([M+Na]^+^), 657.0456; found, 657.0424.

*Data for* **6h**: Yield: 83%, white solid; m.p. 88-90 ^o^C; [α]^20^_D_ = －84 (*c* 3.1 mg/mL, CHCl_3_); IR cm^-1^ (KBr): 3046, 2935, 1789, 1734, 1484, 1407, 1237, 1167, 1023, 781; ^1^H NMR (400 MHz, CDCl_3_) *δ*: 8.02 (d, *J* = 8.4 Hz, 1H), 7.88-7.90 (m, 1H), 7.82-7.85 (m, 1H), 7.46-7.59 (m, 4H), 6.32 (s, 1H, H-5), 6.18 (s, 1H, H-8), 6.01 (s, 1H, H-1), 5.87 (dd, *J* = 9.2, 1.2 Hz, 2H, OCH_2_O), 5.84 (d, *J* = 7.2 Hz, 1H, H-4), 4.53 (dd, *J* = 9.6, 4.0 Hz, 1H, H-11), 4.38 (d, *J* = 9.2 Hz, 1H, H-11), 4.22 (d, *J* = 0.4 Hz, 2H, C_10_H_7_CH_2_), 3.97 (s, 3H, 3′-OCH_3_), 3.94 (s, 3H, 5′-OCH_3_), 3.82 (s, 3H, 4′-OCH_3_), 3.19 (dd, *J* = 7.2, 4.0 Hz, 1H, H-3); ^13^C NMR (100 MHz, CDCl_3_) *δ*: 172.7, 171.7, 149.2, 148.9, 148.1, 147.5, 147.3, 133.9, 133.1, 131.8, 129.7, 128.9, 128.5, 128.3, 127.5, 127.4, 126.6, 126.0, 125.7, 125.5, 123.4, 107.7, 106.0, 101.4, 71.9, 71.2, 64.7, 61.3, 61.2, 61.1, 49.0, 44.0, 39.4; HRMS (ESI): Calcd for C_34_H_27_O_9_Cl_3_Na ([M+Na]^+^), 707.0612; found, 707.0640.

*Data for* **6i**: Yield: 95%, white solid; m.p. 176-178 ^o^C; [α]^20^_D_ = －94 (*c* 3.4 mg/mL, CHCl_3_); IR cm^-1^ (KBr): 3051, 2935, 1785, 1735, 1486, 1228, 1107, 1009, 866; ^1^H NMR (400 MHz, CDCl_3_) *δ*: 6.71 (s, 1H, H-5), 6.63 (s, 1H, H-8), 6.60 (s, 1H, H-6′), 5.931-5.95 (m, 3H, OCH_2_O, H-4), 5.59 (s, 1H, H-1), 4.80-4.81 (m, 2H, H-11), 3.92 (s, 3H, 3′-OCH_3_), 3.88 (s, 3H, 5′-OCH_3_), 3.76 (s, 3H, 4′-OCH_3_), 2.97 (dd, *J* = 5.2, 2.8 Hz, 1H, H-3), 2.15 (s, 3H, COCH_3_); ^13^C NMR (100 MHz, CDCl_3_) *δ*: 171.9, 170.6, 152.7, 150.9, 149.2, 148.2, 142.6, 133.9, 130.9, 123.9, 113.7, 108.76, 108.73, 108.6, 101.7, 75.1, 73.1, 66.8, 61.08, 61.00, 56.2, 49.4, 47.3, 21.0; HRMS (ESI): Calcd for C_24_H_22_O_9_ClBrNa ([M+Na]^+^), 591.0028; found, 591.0028.

*Data for* **6j**: Yield: 93%, white solid; m.p. 169-170 ^o^C; [α]^20^_D_ = －83 (*c* 3.2 mg/mL, CHCl_3_); IR cm^-1^ (KBr): 3074, 2933, 1785, 1733, 1486, 1233, 1171, 1037, 1005, 865; ^1^H NMR (400 MHz, CDCl_3_) *δ*: 6.71 (s, 1H, H-5), 6.62 (s, 1H, H-8), 6.60 (s, 1H, H-6′), 5.930-5.943 (m, 3H, OCH_2_O, H-4), 5.59 (s, 1H, H-1), 4.80-4.81 (m, 2H, H-11), 3.92 (s, 3H, 3′-OCH_3_), 3.88 (s, 3H, 5′-OCH_3_), 3.75 (s, 3H, 4′-OCH_3_), 2.96-2.985 (m, 1H, H-3), 2.35 (q, *J* = 7.6 Hz, 2H, COCH_2_CH_3_), 1.20 (t, *J* = 7.6 Hz, 3H, COCH_2_CH_3_); ^13^C NMR (100 MHz, CDCl_3_) *δ*: 174.2, 171.9, 152.7, 150.9, 149.2, 148.2, 142.7, 134.0, 130.8, 124.1, 113.7, 108.9, 108.7, 108.5, 101.7, 74.8, 73.1, 66.8, 61.08, 61.00, 56.2, 49.4, 47.2, 27.5, 9.0; HRMS (ESI): Calcd for C_25_H_24_O_9_ClBrNa ([M+Na]^+^), 605.0184; found, 605.0169.

*Data for* **6k**: Yield: 98%, white solid; m.p. 88-90 ^o^C; [α]^20^_D_ = －68 (*c* 3.6 mg/mL, CHCl_3_); IR cm^-1^ (KBr): 3062, 2935, 1789, 1736, 1484, 1232, 1172, 1006, 864, 696; ^1^H NMR (400 MHz, CDCl_3_) *δ*: 7.280-7.39 (m, 5H), 6.70 (s, 1H, H-5), 6.59 (s, 1H, H-8), 6.51 (s, 1H, H-6′), 5.937 (d, *J* = 0.8 Hz, 2H, OCH_2_O), 5.88 (d, *J* = 3.2 Hz, 1H, H-4), 5.58 (s, 1H, H-1), 4.78-4.79 (m, 2H, H-11), 3.92 (s, 3H, 3′-OCH_3_), 3.88 (s, 3H, 5′-OCH_3_), 3.75 (s, 3H, 4′-OCH_3_), 3.67 (d, *J* = 7.2 Hz, 2H, PhCH_2_), 2.990 (dd, *J* = 5.2, 3.2 Hz, 1H, H-3); ^13^C NMR (100 MHz, CDCl_3_) *δ*: 171.8, 171.6, 152.7, 151.0, 149.2, 148.2, 142.7, 133.9, 132.6, 130.8, 129.0, 128.9, 127.7, 123.8, 113.8, 108.9, 108.6, 108.3, 101.7, 75.4, 72.9, 66.8, 61.09, 61.01, 56.3, 49.3, 47.2, 41.1; HRMS (ESI): Calcd for C_30_H_26_O_9_ClBrNa ([M+Na]^+^), 667.0341; found, 667.0320.

*Data for* **6l**: Yield: 92%, white solid; m.p. 90-92 ^o^C; [α]^20^_D_ = －67 (*c* 3.7 mg/mL, CHCl_3_); IR cm^-1^ (KBr): 3047, 2933, 1788, 1735, 1484, 1231, 1170, 1034, 779; ^1^H NMR (400 MHz, CDCl_3_) *δ*: 7.90-7.94 (m, 2H), 7.84 (d, *J* = 7.6 Hz, 1H), 7.540-7.57 (m, 2H), 7.39-7.48 (m, 2H), 6.71 (s, 1H, H-5), 6.58 (s, 1H, H-8), 6.43 (s, 1H, H-6′), 5.931 (dd, *J* = 6.0, 1.2 Hz, 2H, OCH_2_O), 5.87 (d, *J* = 3.2 Hz, 1H, H-4), 5.57 (s, 1H, H-1), 4.741 (d, *J* = 2.8 Hz, 2H, H-11), 4.12 (d, *J* = 7.2 Hz, 2H, C_10_H_7_CH_2_), 3.92 (s, 3H, 3′-OCH_3_), 3.88 (s, 3H, 5′-OCH_3_), 3.72 (s, 3H, 4′-OCH_3_), 2.99 (dd, *J* = 6.0, 1.2 Hz, 1H, H-3); ^13^C NMR (100 MHz, CDCl_3_) *δ*: 171.78, 171.75, 152.7, 151.0, 149.2, 148.2, 142.8, 133.98, 133.95, 131.7, 130.7, 129.3, 129.1, 128.7, 127.9, 126.8, 126.1, 125.5, 123.7, 123.0, 113.8, 109.0, 108.6, 108.3, 101.7, 75.4, 72.7, 66.7, 61.08, 61.00, 56.3, 49.2, 47.1, 39.0; HRMS (ESI): Calcd for C_34_H_28_O_9_ClBrNa ([M+Na]^+^), 717.0497; found, 717.0505.

*Data for* **9**: Yield: 52%, white solid, m.p. 264-266 ^o^C(lit^3^. m.p. 268-271 ^o^C); [α]^20^_D_ = 2 (*c* 3.0 mg/mL, CHCl_3_); IR cm^-1^ (KBr): 3067, 2940, 2910, 1764, 1464,1118, 890; ^1^H NMR (500 MHz, CDCl_3_) δ: 7.70 (s, 1H), 7.20 (s, 1H), 7.11 (s, 1H), 6.55 (s, 2H), 6.08 (s, 2H, OCH_2_O), 5.38 (s, 2H), 3.96 (s, 3H, 4′-OCH_3_), 3.84 (s, 6H, 3′, 5′-OCH_3_).

**1.2. General procedure for synthesis of 7a-**c **from** **5a-c.**

To solution of **5a**-**c** (0.15 mmol) in dry DCM (5 mL), a solution of BF_3_·Et_2_O (0.18 mmol) in dry DCM (5 mL) was added dropwise to keep the temperature below -15 °C. After adding, the reaction temperature was raised from -15 °C to r.t., and the reaction process was checked by TLC analysis. When the reaction proceeded for 16-24 h, compounds **5a-c** were disappeared. Finally, the mixture was diluted by DCM (30 mL), washed by water (20 mL), HCl (0.1 mol/L, 20 mL), 5% NaHCO_3_ (20 mL) and brine (20 mL), dried over anhydrous Na_2_SO_4_, concentrated in vacuo, and purified by PTLC to give **7a**-**c** (51-98%) as the white solids.

**1.3. Investigation of 5a not in the presence of BF_3_·Et_2_O.**

A solution of **5a** (0.031 mmol) in dry DCM (5 mL) was stirred at r.t. for 2 weeks, and no new product was checked by TLC.

**1.4. Investigation of 5c in the presence of BF_3_·Et_2_O at the different temperatures.**

To a solution of **5c** (0.057 mmol) in dry DCM (5 mL) at -78 °C, a solution of BF_3_·Et_2_O (0.068 mmol) in dry DCM (2 mL) was added dropwise to keep the temperature below -78 °C. After adding, the reaction was maintained at this temperature for 5.5 h, there was no new product checked by TLC. Similarly, at -40 °C for 20 h, or -15 °C for 24 h, no new product was detected.

**1.5. Investigation of 5c in the presence of BF_3_·Et_2_O at the different temperatures.**

A solution of **5c** (0.057 mmol) and AlCl_3_ (0.068 mmol) in dry DCM (5 mL) was cooled at -15 °C for 20 min, then the reaction temperature was raised from -15 °C to r.t. for 24 h. Finally, the mixture was concentrated in vacuo, and purified by PTLC to give **7c** in 77% yield as a white solid.

**1.6. Investigation of 8a in the presence of BF_3_·Et_2_O.**

To a solution of **8a** (0.145 mmol) in dry DCM (5 mL) at -15 °C, a solution of BF_3_·Et_2_O (0.174 mmol) in dry DCM (5 mL) was added dropwise to keep the temperature below -15 °C. After adding, the reaction temperature was raised from -15 °C to r.t. When the reaction proceeded for 48 h, the mixture was diluted by DCM (30 mL), washed by water (20 mL), HCl (0.1 mol/L, 20 mL), 5% NaHCO_3_ (20 mL) and brine (20 mL), dried over anhydrous Na_2_SO_4_, concentrated in vacuo, and purified by PTLC to give **9** in 52% yield as a white solid.

**1.7. Investigation of 8b in the presence of BF_3_·Et_2_O.**

To a solution of **8b** (0.15 mmol) in dry DCM (5 mL) -15 °C, a solution of BF_3_·Et_2_O (0.18 mmol) in dry DCM (5 mL) was added dropwise to keep the temperature below -15 °C. After adding, the reaction temperature was raised from -15 °C to 9 °C. If the reaction proceeded for 24 h, there was no new product checked by TLC. Then the mixture was reacted at 40°C for 48 h, no new product was detected.

**References:**

(1) Petcher, T. J.; Weber, H. P.; Kuhn, M.; von Wartburg. A. *J. Chem. Soc*. **1973**, *3*, 288－292.

(2) Xu, H.; Xiao, X.; Wang, Q. T. *Bioorg. Med. Chem. Lett*. **2010**, *20*, 5009−5012.

(3) Nishii, Y.; Yoshida, T.; Asano, H.; Wakasugi, K. et al. *J. Org. Chem*. **2005**, *70*, 2667-2678.

**2. Comparison of 6e-h and 6e′-h′ about NMR spectra**

**6e** (^1^H NMR)

**6e′** (^1^H NMR)

6e (^13^C NMR)

**6e′** (^13^C NMR)

**6f** (^1^H NMR)

**6f′** (^1^H NMR)

**6f** (^13^C NMR)

**6f′** (^13^C NMR)

**6g** (^1^H NMR)

**6g′** (^1^H NMR)

**6g** (^13^C NMR)

**6g′** (^13^C NMR)

**6h** (^1^H NMR)

**6h′** (^1^H NMR)

**6h** (^13^C NMR)

**6h′** (^13^C NMR)

**3. Copies of ^1^H NMR and ^13^C NMR spectra**


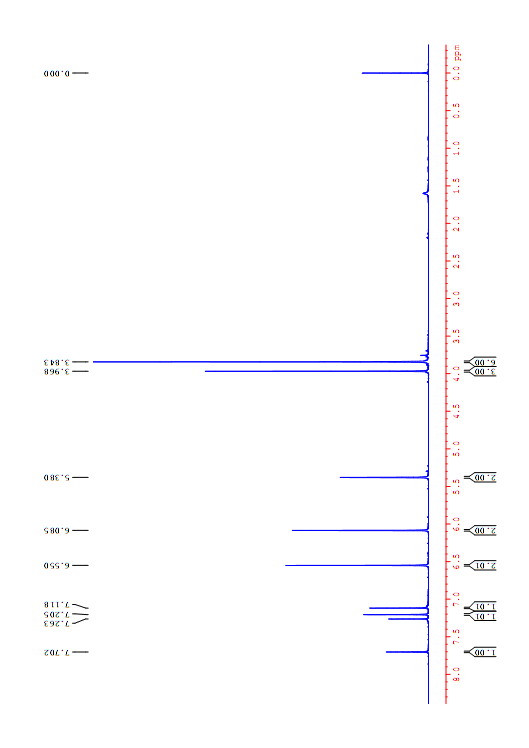

Supplement: Supplementary Information [file srep16285-s1.doc]
